# Does social motivation mitigate fear caused by a sudden sound in horses?

**DOI:** 10.1007/s10071-023-01805-x

**Published:** 2023-07-14

**Authors:** Wiktoria Janicka, Izabela Wilk, Tomasz Próchniak

**Affiliations:** 1 Department of Horse Breeding and Use, University of Life Sciences in Lublin, 13 Akademicka Street, Lublin, Poland; 2 Institute of Biological Basis of Animal Production, University of Life Sciences in Lublin, 13 Akademicka Street, Lublin, Poland

**Keywords:** Horse, Gregariousness, Social dependence, Fearfulness, Social buffer

## Abstract

Living in a herd has multiple advantages for social species and is a primary survival strategy for prey. The presence of conspecifics, identified as a social buffer, may mitigate the individual stress response. Social isolation is, therefore, particularly stressful for horses, which are gregarious animals. However, they are not equally vulnerable to separation from the group. We tested whether more and less socially dependent horses and independent individuals would differ in their responses to novel and sudden sounds occurring in two contexts: non-social and social motivation. Twenty warmblood horses were first exposed to two social tests: to evaluate the level of social dependence (rate of restless behaviour; social isolation) and the quantity and the quality of interactions in which they were involved (stay on a paddock). Two fear audio tests were then performed to compare the responses to sudden sounds while feeding (non-social motivation; control trial) and while moving towards the herd (social motivation; experimental trial). Socially dependent horses showed more pronounced avoidance behaviour and needed much more time to resume feeding during the control trial. Hence, dependent individuals appeared to be more fearful. However, during an experimental trial, horses of both groups tended to ignore the sound or paid only limited attention to the stimulus, continuing to move forward towards their conspecifics. Thus, social motivation may mitigate fear caused by a frightening stimulus and make fearful and dependent horses more prone to face a potentially stressful event. This finding should be taken into account in horse training and management.

## Introduction

Horses’ reactivity to different stressors and environmental stimuli is modified by the animal’s motivation, fearfulness or curiosity (Kozak et al. 2018). The variety of these responses may stem from differences in coping styles or temperamental traits (Visser et al. 2002) and has major implications in animal husbandry (Rothmann et al. 2014). Responding to suddenness and novelty may compromise both the safety of the handler and the welfare of a horse (Acton et al. 2020; Vidament et al. 2021). It is, therefore, crucial to be aware of the possible reactions of the horses (Corgan et al. 2021). Temperament is defined as any characteristic of an individual that appears to be stable over both time and situation (Roberts et al. 2016). Fearfulness and gregariousness, i.e. reactivity to social isolation from conspecifics (Lansade et al. 2008b), are among the most well-known and particularly interesting dimensions of equine temperament (Lansade et al. 2008a, 2017; Górecka-Bruzda et al. 2022). Indeed, these traits relate directly to the evolution of the horse, which is a highly social prey animal that lives in a herd and remains in constant readiness to detect and avoid the threat (Marliani et al. 2021).

A stable and organised social structure, manifested by dominance and submissive interactions, enables horses to avoid the deleterious energy and health costs that would be incurred in aggressive encounters (Narciso et al. 2021). The group members help to improve foraging strategies, provides learning opportunities (Krueger and Heinze 2008; Krueger et al. 2014; Mendonça et al. 2021) and facilitates localising and avoiding a potential threat (Hothersall and Casey 2012). For social species, living in a herd is the primary antipredator defence mechanism (Apfelbach et al. 2005). Although predation pressure is relaxed in domestic horses (Janczarek et al. 2021), they experience multiple anthropogenic stressors (Squibb et al. 2018). According to the risk-disturbance hypothesis, various sounds, objects and events may trigger a response analogous to the presence of a predator (Frid and Dill 2002). In this context, social support from other members of the herd consists of social buffering that mitigates individual stress levels (Hartmann et al. 2012).

When the alternative is no possibility of social interaction, horses are highly motivated to make any kind of physical contact (full, head or muzzle) with other individuals (Søndergaard et al. 2011). They strengthen relationships through affiliative approaches, mutual grooming or keeping proximity (Wolter et al. 2018). However, if direct contact is not possible, they at least strive for indirect interactions (Yarnell et al. 2015). For horses, social isolation is a particularly strong stressor that negatively affects their behavioural and physiological reactivity (Kay and Hall 2009; Żelazna and Jezierski 2018). When returning to the group after a previous restriction of social opportunities, horses may manifest a rebound effect, which refers to increased intensity of interactions with conspecifics (Christensen et al. 2002a). Stress elicited by social isolation may even moderate mild somatic pain (Reid et al. 2017). However, horses do not respond to the separation from the group with the same strength (Wolff et al. 1997). Socially dependent individuals have difficulty functioning when out of the herd. In contrast, independent animals are not excessively bonded to other horses or humans and are self-reliant when left away from the herd (Burattini et al. 2020). In the previous study (Janicka et al. 2022a), we showed that the motivation for social interaction rapidly decreased the potential of a self-applied acoustic stimulus in creating virtual barriers. Only 20% of horses turned back or stopped after the playback of acute alarming sound (sudden, unexpected) when the second horse was present in the excluded zone. In turn, when food reward was offered in that zone, the virtual barrier was 80% effective and made the horses respond with flight, going away or stopping.

Here, we assessed if horses characterised by different levels of social dependence would differ in their fear reaction to the sudden sounds occurring in two different contexts: social (while moving towards the herd) and non-social (while consuming attractive food). Given the significance of the social nature of horses, we hypothesised that socially dependent and independent individuals may show different fear responses in the non-social, but similar and weak responses in the social context, which would highlight the importance of social motivation in reducing the effect of frightening stimulus.

## Materials and methods

### Ethical statement

All experimental procedures were carried out in accordance with the European directive 2010/63/EU and Polish laws—the Act of January 23, 2021—Law on the organization of breeding and reproduction of farm animals (Journal of Laws 2021, Item 36) and were approved by the Local Ethics Review Committee for Animal Experiments (no 58/2022), Lublin, Poland.

### Animals and living conditions

The study involved 20 adult warmblood horses (aged 6–16 years; 10.6 ± 3.6 years), comprising ten mares and ten geldings. The horses knew each other as they had been housed in the same equestrian centre for at least two years. They were released to an extensive pasture or occasionally to several paddocks for about six hours a day, where they could engage in social interaction. All of the subjects were familiar with the paddocks and pastures used in the study. Thus, habituation to the environment was not necessary.

The subjects were kept in individual box stalls bedded with wheat straw and equipped with a manger, hay feeder, salt lick and an automatic waterer. They were fed three times a day with a mixture of oats and bran and had unlimited access to hay. The horses worked under the saddle for a maximum of two hours a day, six days a week. The subjects were under permanent veterinary control (periodic physical examination, assessment during the movement, anamnesis, monitoring heart rate) and were regularly observed by an experienced caretaker during daily handling. Additionally, they were examined one week before and after the study (the above procedures). No physical or behavioural disorders were found before or after the study.

The tests were carried out on calm, windless days (< 0.3 m/s) during the spring period of 2022 and 2023. The location of the testing area reduced unnecessary stimuli which could interfere with the perception of the sound stimulus and affect horse’s reactions, including auditory stimuli, movement of vehicles, pedestrians, or other horses. No sudden and loud noises (e.g. dog barking, tractor noise, or other equipment) interrupted the testing procedures.

Two adult female experimenters handled the horses during the study. They were known to the horses, as they were also their caretakers and looked after them five days a week.

### Social tests

Prior to the main part of the experiment (Sound tests), the horses were subjected to two different social tests to assess the level of social dependence and engagement in social interactions. The tests were conducted on separate, consecutive days.

#### Social dependence test–arena test

Dependence on the other members of the social group was assessed during the herd (social) dependence test. The test consisted of separation from a herd that stayed on the pasture about 300 m from a tested subject. Horses were individually released to a small paddock (400 m^2^) and were observed for a subsequent five minutes using the focal animal sample method (Altmann 1974). This time appeared to be sufficient because individual differences in reactions to an unusual situation were shown to be the strongest within the first few minutes (Wolff et al. 1997).

Behaviours that were assessed during the test were chosen based on the ethogram used in the arena test by Seaman et al. (2002), but some modifications were implemented. The most frequent behavioural variables indicating negative emotional arousal during isolation were vocalisation, defecation, locomotion and vigilance (Lansade et al. 2008b). Thus, certain behaviours assessed by Seaman et al. (2002) (standing, investigation, tail posture, paw, urination) were waived and the traits: ‘canter’ and ‘snoring’ (instead of snorts produced in positive context (Laurijs et al. 2021)) were added to the ethogram used in the current study. Behaviours assessed as frequency (whinny, snoring and defecation) were primarily recorded. However, since in the current study they were displayed only rarely by a few horses, they were not included in the Table 1 and were not considered when dividing horses into groups with different levels of social dependence.

**Table 1:** Behaviours used to assess social dependence during herd dependence test (modified ethogram worked out based on Seaman et al. (2002))

Behaviour	Description
Sustained walk (s)	Walking energetically, looking in front or around
Trot (s)	A two-beat gait
Canter (s)	^a^A three-beat gait
Vigilance (s)	Standing still with an elevated neck, intently orientated head and ears

^a^Added behaviour (modification of the ethogram)

After conducting and analysing the data from the social dependence test, horses were divided into three groups of social dependence. The level of social dependence was determined on the self-established percentage time of displaying restless behaviours based on variation of horses’ responses:

- independent (I): < 15% (n = 9) of the observation time was accounted for by restless behaviour–horses able to function without the social support of conspecifics (Stachurska et al. 2021),
- moderately dependent (MD): 15–50% (n = 5),
- dependent (D): > 50% (n = 6)–horses not self-reliant, reactive to social isolation from conspecifics (Burattini et al. 2020).

#### Social interaction test–introduction to a paddock

To determine the engagement in social interactions, the horses were observed just after being released into a large paddock (5000 m^2^) in the morning (8.00 a. m.). The procedure was repeated on three consecutive days, with a single observation session lasting ten minutes. This observation time was taken from the study of Seaman et al. (2002), who assessed the dominant and submissive behavioural responses of horses after reintroduction to the group.

Affiliative and agonistic interactions (McDonnell 2003) (Table 2) in which each focal animal was involved were recorded every 30 s using the one-zero sampling method (Altmann 1974). The total number of affiliative, aggressive/threatening and submissive behaviours exhibited by each horse was then calculated. The measurement of spatial proximity (horses standing with body contact or within two horse-lengths), commonly used to assess social bonds (Wolter et al. 2018), was not taken into account because of the short-term character of the social test, whereas horses show a mean latency of changing the spatial distribution of group members every 8 min (Christensen et al. 2002b).

**Table 2:** Behaviours recorded during social interaction test (McDonnell 2003)

Affiliative behaviour	Aggressive and threatening behaviour
Mutual grooming (nipping/nuzzling/rubbing each other’s neck, mane, chest, back, rump or tail) Laying the head on the neck, back or rump Approach (forward movement toward another horse and staying close) Following (moving along behind another horse, no attempt to attack) Playing with conspecifics (any reaction or sequence of behaviour during play in motion, occurring socially)	Head, bite or kick threat Bite, kick Head bump Strike (one or both forelegs rapidly extended forward to contact another horse, often accompanied by a squeal or snort) Rear (raising of the forelegs into the air, supporting the body on the hind legs only) Herding (combination of head threat and ears laid back with forward locomotion) Chase (pursuit of another horse, usually at a gallop)

Submissive behaviour–avoidance of aggressive/threatening interaction

### Sound tests

Sound tests were the startling fearfulness tests, assessing horses’ reactions to a sudden auditory stimulus (Seaman et al. 2002). However, these tests were conducted in two different contexts: without (a control trial) and with (an experimental trial) a social motivation. Each sound test was carried out twice (two stages) with an interval of about a year (to reduce the likelihood of habituation to the sounds). Two different sounds were used during these tests: sound A (squeal of a pig, produced during distress (Çavuşoğlu et al. 2020)) and sound B (futuristic characteristics; similarity to radio and TV interference, crackling). The sounds were selected based on the results of our previous studies: comparing reaction to 40 sounds of different origin (Janicka et al. 2022a), assessing the possibility to use sound stimulus in creating virtual barrier (Janicka et al. 2022b) and comparing reactions to 40 known and four novel sounds (unpublished data). Sounds that were chosen elicited similar and strongest avoidance behaviour. Both sounds were unknown to the study horses. In the first stage of the study (stage 1; 2022 year), sound A was played during the control trial and sound B during the experimental trial. The reverse order was then used in the second stage (stage 2; 2023 year). In addition, 10 randomly selected horses were first subjected to the experimental and then to the control trial and for the remaining 10 horses, the order of procedures was reversed. The experimental trial was conducted in the morning (7.30–10.30) when horses were normally released to the extensive pasture to induce the motivation to move forward and to join the herd. In turn, the control trial was carried out in the afternoon/evening hours (15.30–19.30) when horses were brought back to the stable and were used to stay in boxes or paddocks near the stable (reduction of social motivation). The interval between two trials was at least six days (6–8 days; differences caused by poor weather conditions: rain or wind > 0.3 m/s).

#### Non-social motivation sound test (NMST)–a control trial

The horses were individually subjected to a controlled assessment of their fear response to a new and unexpected sound while eating (non-social motivation sound test; NMST). The testing arena (140 × 30 m) was designated with tape for an electric fence on one of the known paddocks. It was located next to another paddock, providing proximity (approximately 50 m) to conspecifics during the test to avoid the impact of social isolation stress and lack of concentration on the food provided. The three horses in the neighbouring paddock (the same animals throughout the experiment, visible to study subjects) were habituated to the test sound a few days before the study.

A plastic container for the later provision of attractive food was placed at the front of the testing arena. A loudspeaker (JBL Charge 4, rated power of 30W) was positioned 1 m in front of the container, allowing the horse a long escape distance after the sound playback, if necessary. Prior to the test, horses were taught to feed from the container.

Each horse was released into a testing arena 15 min before the start of the test to habituate to the experimental setting. The first experimenter (E1) then brought the horse to the start point (3 m from the container) and waited for the test to begin. At this time, the second experimenter (E2) put chopped carrots inside the container. The horse was unleashed (by E1) and could start feeding, and both experimenters left the arena. After 30 s, E2 played a sudden sound (20 s, 40 dB; first stage–sound A, second stage–sound B) via Bluetooth by a Samsung Galaxy A02s device (Samsung Electronics Co., Ltd., South Korea). As the maximum range of Bluetooth was approximately 15 m, experimenters had to stay at this distance during the test in order to play the recording at the right moment. However, they stood calmly, did not disrupt the test and the horses were used to their presence (they were also their caretakers).

Latency to respond to the stimulus was proved to be the objective and easy measure of horses’ responsiveness in fear tests (Górecka-Bruzda et al. 2011). Therefore, latency to resume feeding (s) was measured in the current study. If a horse did not approach the food within 300 s, it was given 301 s (Górecka-Bruzda et al. 2011). Additionally, a 5–point behavioural scale describing horses’ reactions after sound playback was developed. Receiving 5 points indicated the highest level of fear (Table 3).

**Table 3:** Behavioural scale used to assess horses’ responsiveness during the sudden sound test

Points	Behavioural response
1	The horse pays no attention to the sound, continues to take food in an uninterrupted manner and is calm
2	The horse raises its head, puts up its ears and resumes feeding. It is not spooked, and it does not walk away from the food
3	The horse lifts its head, ears up, walks away a few steps, looks at it, approaches cautiously and starts to eat, may snoring
4	The horse abruptly removes its head and jumps back. Spins, impatient, may toss head, hesitantly approaches food, still spooked
5	The horse abruptly jumps back, walks away/runs a considerable distance, snorts, is afraid to approach food for a very long time (hesitant, tense) or does not approach at all

#### Social motivation sound test (SMST)–an experimental test

The social motivation sound test (SMST) was a modified version of the sound tunnel test developed in the previous study (Janicka et al. 2022a) to assess the potential of the sounds in creating electric-free virtual fences. During the test, the horses moved through the same corridor (55 m length, 5 m width, designated with tape for an electric fence) leading towards a known pasture, where the other members of the herd (except for individuals included in the study) were staying. The start–end of the corridor was closed after the introduction of a horse to a start zone (first 5 m), whereas the finish-end was permanently open, allowing the animal to leave it after each run. The experimental paddock, at which the corridor was designated, was separated from the pasture by metal railings. There was a loudspeaker placed outside the corridor at the finish line. The day before the SMST, horses were brought into the experimental paddock for two hours to familiarise themselves with the corridor.

SMST was conducted in the morning hours on three consecutive days (three trials). During each trial, horses went through the corridor at least three times: (1) being led by the experimenter (E1), (2) freely, without the experimenter (E1), and (3) freely, but with playing a sudden sound by E2 (20 s, 100 dB; first stage–sound B, second stage–sound A) when the individual reached a distance of 10 m from the finish line. The initial two trials evaluated the horse's willingness to walk towards the pasture and other horses located approximately 150 m away. If the horse exhibited this behaviour, a third trial was conducted to observe its reactions. Before each trial, the study subject was held on the line in a start zone by E1. The second experimenter (E2) waited in a distance of 15 m from the loudspeaker to play the sound at the right moment after the horse was released by E1.

We showed that the motivation for social interaction rapidly decreased the audio virtual fencing effect (Janicka et al. 2022a). Thus, we focused on subtle differences in reaction to the sound between horses with different levels of social characteristics. The sound barrier effect, completion of the SMST and behaviour after completion of the test were recorded (Table 4).

**Table 4:** Behavioural variables assessed during social motivation sound test

Behaviour	Description
Barrier effect (scale 1–5)	Reaction to the sound; 1–continuing forward movement, 2–slowing down, 3–stopping, 4–going away, 5–running away
Completion of the test (scale 1–2)	Leaving a corridor before the end of the 20 s sound; 1–no, 2–yes
Finish behaviour (scale 1–6)	Behaviour after completion of the test; 1–free walking/resting near the finish line; 2–free walking/resting away from the finish line, 3–looking around nervously + no locomotion, 4–walking + looking around nervously, 5–trot/canter + looking around nervously, 6–not reaching a finish line within 20 s of the sound

### Statistical analyses

The statistical analysis was performed with the SAS 9.4 software (version 9.4, SAS Institute Inc., Cary, NC, USA). Since the data did not conform to a normal distribution, as determined by the Kolmogorov–Smirnov and Shapiro–Wilk tests (p < 0.05), non-parametric tests were applied. Correlations between the results of behavioural tests were checked with + Spearman's rank correlation. Kruskal–Wallis test was used to evaluate if social dependence affected the horses’ responses to the sounds and engagement in affiliative and dominant-submissive interactions. Dwass-Steel-Critchlow-Fligner (DSCF) multiple comparisons were then performed to check for the significance of differences between the groups. Additionally, the differences between mares (n = 10) and geldings (n = 10) and between younger (n = 10; 6 – 10 years) and older horses (n = 10; 11–16 years) during the tests (Mann–Whitney U test), as well as between two stages of the study (Wilcoxon test) were checked. Even though the significance of results was calculated via non-parametric tests, for better clarity and readability for readers, the results were presented in tables as mean value ± standard error of the mean (SE). The minimum level of significance for all tests used was accepted at p < 0.05.

## Results

### Stage of the study differences

No significant differences were found between the results of sound tests in two stages of the study (the revised order of sounds used in the control and the experimental trial) (p > 0.05) (Table 5). Horses responded similarly, no matter which sound (A–squeal of a pig, or B–futuristic sound) was used as control or experimental. For this reason, further comparisons between the reaction in two stages were waived.

**Table 5:** Comparison between reactions in social and non-social sound tests in dependence of the stage of the study

Variable	Stage 1	Stage 2
Latency to resume feeding in NMST (s)	104.45 ± 16.97 a	133.75 ± 16.31 a
Behavioural scale in NMST (1–5)	3.60 ± 0.18 a	2.90 ± 0.15 a
Barrier effect in SMST (1–5)	3.05 ± 0.17 a	3.45 ± 0.17 a
Completion of the SMST (1–2)	1.82 ± 0.05 a	1.78 ± 0.05 a
Finish behaviour in SMST (1–6)	2.50 ± 0.25 a	2.17 ± 0.21 a

Means (± SE) marked with different letters differ significantly between stage 1 and stage 2 of the study at p < 0.05 (Wilcoxon test)

*NMST* non-social motivation sound test, *SMST* social motivation sound test, *stage 1* squeal of a pig (sound A) as control sound and futuristic sound (sound B) as experimental sound, *stage 2* changed order of the sounds

### Dependence on the social group and need for social interactions

Most of the horses (n = 11) showed more (n = 6; dependent) or lesser (n = 5; moderately dependent) signs of anxiety when they were physically isolated from the group (Table 6). Of all the other horses, those categorised as dependent spent the most time demonstrating a sustained walk (DSCF = 3.8730, p = 0.0170; DSCF = 5.0767, p = 0.0010; in comparison to moderately dependent and independent, respectively). The time of vigilance was similar in the dependent and moderately dependent groups (DSCF = 0.2582, p = 0.9818) and definitely longer than in independent horses (DSCF = 4.1779, p = 0.0088; DSCF = 4.2567, p = 0.0074, respectively). Canter rarely occurred and did not differ significantly between the groups (p > 0.05). Dependent horses trotted (DSCF = 3.9485, p = 0.0145) more than independent horses but not moderately dependent individuals (DSCF = 2.7749, p = 0.1217; DSCF = 2.9863, p = 0.0875, respectively).

**Table 6:** Comparison of the time budget of horses with different levels of social dependence based on the social dependence test

Behaviour	Dependent (N = 6)	Moderately dependent (N = 5)	Independent (N = 9)
Time budget			
Sustained walk (s)	188.67 ± 22.04 c	34.00 ± 7.31 b	0.00 ± 0.00 a
Trot (s)	23.33 ± 8.44 b	3.60 ± 2.91 ab	1.22 ± 1.22 a
Canter (s)	2.67 ± 1.26 a	2.40 ± 1.47 a	0.56 ± 0.55 a
Vigilance (s)	45.83 ± 10.36 b	46.00 ± 13.85 b	9.44 ± 3.02 a
Frequency of social interactions			
Affiliative interactions	23.67 ± 3.29 a	24.80 ± 4.69 a	19.11 ± 4.34 a
Dominance behaviour	4.17 ± 1.92 a	2.00 ± 0.71 a	4.22 ± 1.14 a
Submissive behaviour	3.83 ± 0.54 a	2.00 ± 0.71 a	1.88 ± 0.35 a

Means (± SE) marked with different letters differ significantly in rows at p < 0.05 (DSCF)

Although horses responded differently to social isolation, the quantity and the quality of interactions during their stay on a paddock (social interaction test) were similar between the groups (p > 0.05). The horses usually displayed affiliative interactions, whereas dominance and submissive behaviours were rarely observed. For this reason, agonistic behaviours were not included in further analysis.

### Reactions to sounds

All the horses (n = 20) moved willingly towards the pasture in SMST in the first two trials and were subjected to the third trial (the proper test), in which their reactions were assessed.

Horses’ responses to a sudden sound in NMST varied, but they generally interrupted feeding for some time (stage 1; n = 17, stage 2; n = 18) and showed movement in the opposite direction (stage 1; n = 13, stage 2; n = 16). In contrast, they rarely stopped forward movement (stage 1; n = 4, stage 2; n = 5) and turned back towards the start zone (stage 1; n = 2, stage 2; n = 1) in SMST. Their reactions were usually limited to slowing down after hearing a sound while moving towards the other herd members.

Dependent horses were more fearful during NMST than independent horses (Table 7). They needed much more time to resume feeding after the sound was played (DSCF = 6.0376, p < 0.0001) and had higher scores on the behavioural scale, indicating the strength of the fear response (DSCF = 6.3439, p < 0.0001). In spite of this, no differences were found between the groups during the SMST (p > 0.05). The sound barrier effect was poor and usually limited to slowing down or stopping for a while, and eventually, most of the horses finished the test.

**Table 7:** Impact of the social dependence of the horses on reactions to sounds

Behaviour	Social characteristics of the horses		
	Dependent (N = 6)	Moderately dependent (N = 5)	Independent (N = 9)
Sound barier (SMST)	2.19 ± 0.20 a	1.80 ± 0.19 a	1.98 ± 0.16 a
Completion of SMST	1.81 ± 0.07 a	1.83 ± 0.07 a	1.78 ± 0.06 a
Finish behaviour (SMST)	2.64 ± 0.27 a	2.00 ± 0.30 a	2.41 ± 0.27 a
Refeeding latency (SST)	196.75 ± 20.69 b	129.30 ± 26.40 ab	61.67 ± 12.43 a
Behavioural scale (SST)	3.92 ± 0.18 b	3.30 ± 0.33 ab	2.78 ± 0.13 a

Means (± SE) marked with different letters differ significantly in rows at p < 0.05 (DSCF)

There were no correlations between the horses’ reactions in two different sound tests, except for the first reaction after the sound playback (behavioural scale) and latency to refeed in NMST and barrier effect in SMST (Table 8). In the case of the horses that gained more points on the behavioural scale (stronger fear response) and needed more time to resume feeding, the sound barrier effect was greater (Spearman's rank correlation; r_s_ = 0.350, p < 0.0001 and r_s_ = 0.325, p = 0.0003, respectively). However, these correlations were weak.

**Table 8:** Correlations between the results of behavioural tests

Sudden sound test and social tests	Sudden sound test, herd dependence test and social motivation sound test					
	Latency (NMST)	Behav. (NMST)	Restles.	Barrier (SMST)	Compl. (SMST)	Finish (SMST)
Latency (NMST)	−	−	0.354*	0.325*	−0.128	0.149
Behav. (NMST)	−	−	0.397*	0.350*	−0.170	0.063
Restles	−	−	−	0.060	0.040	0.146
Affiliative behaviour	0.315*	0.491*	0.172	0.416*	−0.350*	0.058

^*^Statistically significant correlations (in all cases p < 0.001)

*Latency (NMST)* refeeding latency in NMST, *behav. (NMST)* behavioural scale in NMST, *restless.* restless behaviour (%) in herd dependence test, *barrier (SMST)* effect of sound barrier in SMST, *compl. (SMST)* completion of SMST, *finish (SMST)* finish behaviour in SMST, *NMST* non-social motivation sound test, *SMST* social motivation sound test

Both refeeding latency (r_s_ = 0.354, p < 0.0001) and behavioural scale scoring (r_s_ = 0.397, p < 0.0001) were weakly correlated with the time spent on restless behaviour in the herd dependence test. Simultaneously, restlessness (indicating the level of herd dependence) and the responses during SMST were not correlated (p > 0.05).

There was no correlation between the frequency of affiliative interactions and social dependence (restlessness) (r_s_ = 0.172, p = 0.1898). However, in the case of the horses that interacted more, the sound barrier effect was stronger (more points in the behavioural scale) (r_s_ = 0.416, p < 0.0001) and the horses finished the test with a lower probability (r_s_ = -0.349, p < 0.0001). They also received more points on the behavioural scale during NMST (r_s_ = 0.491, p < 0.0001) and had longer refeeding latencies (r_s_ = 0.315, p = 0.0005).

### Sex and age differences

Mares were more restless when separated from the group during the herd dependence test (Mann–Whitney U test; U = 256.50, p = 0.0037), needed more time to resume feeding after sound playback in NMST (U = 1156.50, p = 0.0007) and responded to this sound more violently (they had a higher score on the behavioural scale) (U = 1260.00, p = 0.0046) than geldings (Table 9). Showing affiliative interactions was common and comparable (U = 427.50, p = 0.7412). Although mares showed submissive behaviour more often (U = 297.00, p = 0.0234), agonistic interactions were rarely presented. No differences between mares and geldings were found in response to the sound during SMST (p > 0.05). The sound barrier effect (stopping/limiting further movement) was weak, and both mares and geldings finished the test with a similar frequency. The only difference concerned behaviour after completion of the SMST. Mares were more agitated while waiting to be reunited with the herd (U = 1030.00, p < 0.0001).

**Table 9:** Sex and age differences during social and sound tests

Variable	Horse groups (sex/age)	
	Mare	Gelding
Restless behaviour (%)	45.37 ± 6.78 b	24.07 ± 5.71 a
Affiliative interactions (freq.)	21.50 ± 1.72 a	22.30 ± 2.19 a
Dominance behaviour (freq.)	3.50 ± 0.67 a	3.80 ± 0.60 a
Submissive behaviour (freq.)	3.00 ± 0.29 b	2.00 ± 0.22 a
Latency to resume feeding in NMST (s)	167.50 ± 17.48 b	70.70 ± 13.27 a
Behavioural scale in NMST (1–5)	3.60 ± 0.18 b	2.90 ± 0.15 a
Barrier effect in SMST (1–5)	2.20 ± 0.16 a	1.80 ± 0.14 a
Completion of the SMST (1–2)	1.78 ± 0.05 a	1.82 ± 0.05 a
Finish behaviour in SMST (1–6)	2.83 ± 0.23 a	1.83 ± 0.21 b

	Younger (6–10 years)	Older (11–16 years)
Restless behaviour (%)	35.50 ± 4.36 a	33.93 ± 4.83 a
Affiliative interactions (freq.)	25.20 ± 1.19 b	18.60 ± 1.42 a
Dominance behaviour (freq.)	3.20 ± 0.31 a	4.10 ± 0.54 a
Submissive behaviour (freq.)	2.60 ± 0.19 a	2.40 ± 0.19 a
Latency to resume feeding in NMST (s)	99.0 ± 15.73 a	139.20 ± 17.32 a
Behavioural scale in NMST (1–5)	3.30 ± 0.18 a	3.20 ± 0.17 a
Barrier effect in SMST (1–5)	2.05 ± 0.15 a	1.95 ± 0.15 a
Completion of the SMST (1–2)	1.75 ± 0.06 a	1.85 ± 0.05 a
Finish behaviour in SMST (1–6)	2.45 ± 0.23 a	2.22 ± 0.23 a

Means (± SE) marked with different letters differ significantly between mares and geldings or younger and older horses at p < 0.01 (Mann–Whitney U test)

*NMST* non-social motivation sound test, *SMST* social motivation sound test

There were no differences between the responses of younger and older horses during the herd dependence test, NMST and SMST (p > 0.05). Only during the social interaction test younger individuals showed significantly more affiliative interactions (U = 1134.00, p = 0.0005).

## Discussion

Horses evolved as prey species for which living in a herd is a survival strategy (Marliani et al. 2021). Thus, isolation from conspecific is a particularly strong stressor for horses (Kay and Hall 2009). However, similarly to Wolff et al. (1997), Lansade et al. (2008b) and Górecka-Bruzda et al. (2022), we showed that they were not equally vulnerable to separation from the herd. The differences in isolation anxiety were mainly displayed by various engagement in sustained walking, vigilance and trotting, whereas neighing, snorting and defecation were infrequent. In turn, Lansade et al. (2008b) point to the frequency of neighing as the most interesting behavioural parameter, which was well correlated with other parameters measured in the same isolation tests, such as defecation, locomotion and vigilance. The differences in these observations may be due to the individual characteristics of the horses. Also some possibility of auditory contact between the study subjects and the herd (staying in a distance of 300 m) can not be entirely excluded, which is a limitation of our study. Besides the existence of a trait ‘social dependence’, the isolation test also revealed the importance of the factor ‘sex’ on gregariousness. We noticed that, compared to geldings, mares were more restless when separated from the herd. This observation is in agreement with the study of Górecka-Bruzda et al. (2022), who showed that mares were more socially dependent than geldings. The current study further showed that social dependence is not manifested by engagement in social interactions. After the introduction to a large paddock during the social interaction test, the horses presented a wide range of affiliative interactions, including mutual grooming, following, approach and motor playing. The frequency of affiliative relationships did not vary between horses of different levels of gregariousness, or between mares and geldings. The similar engagement in interactions of horses with different social characteristics stresses the importance of social contact with conspecifics. Additionally, the fact that horses with different levels of gregariousness made a similar number of social interactions indicates that correlation between engagement in affiliative interactions and higher agitation after sound playback both in a non-social and social motivation context were due to greater fearfulness rather than social dependence. We further confirmed that social dependence does not result from the need for direct interactions but rather from the very need to stay close to the herd and is best observed during social isolation (Le Scolan et al. 1997; Wolff et al. 1997; Seaman et al. 2002; Lansade et al. 2008b; Lansade and Simon 2010). However, we did not consider spatial proximity between the horses in the social interaction test, which, as one of the social bond measurements, could show correlations with social dependence. It would be worthwhile to include this trait in future research. In the current study agonistic interactions were rare and limited exclusively to threatening, so it was not possible to compare such behaviour in dependent and independent individuals. Similarly, Kimura (1998) noticed that during spring season, thus, when breeding season starts, dominance–submission interactions were not frequent between free-ranging mares. We also observed horses in spring, but in the case of our study the results could stem from a short period of observation. Furthermore, unlike Kimura’s (1998) study, our experiment was not conducted at different seasons of the year. The limitation of social interaction test after introduction to a paddock (even if simple and easy to repeat) in assessing the full range of equine social behaviour should be taken into account in further studies.

As we predicted, horses of different levels of social dependence varied in their responses to the sudden sound during the test in a non-social (but not a social) motivation context when they moved towards a herd on a pasture. Horses classified as dependent were more fearful compared to independent individuals in a non-social motivation context. They responded to the sound more violently (jumped back and walked/run away more often) and needed significantly more time to resume feeding than self-reliant animals. Briard et al. (2015) also noted that fearless horses were simultaneously less gregarious. In turn, Villalba et al. (2009) showed that sheep cautious in accepting new food were more reactive during social isolation. Indeed, in many species, these traits have been used to characterise ‘bold’ and ‘shy’ behavioural types (Briard et al. 2015). We found no correlations between the rate of restlessness during separation from the herd and the results of the social motivation sound test. Horses’ reactions were usually limited to slowing down after hearing the sound or eventually to stopping for a while. Even when hesitating, they decided to move forward to stay closer to the herd. Thus, the motivation to join the group was too strong for an unexpected, novel sound to stop both independent and dependent horses from further movement. Jouven et al. (2012) showed that some of the sheep, previously trained not to exceed the virtual fence, followed their conspecifics regardless of the electric shocks they received. Since, in our study, dependent and moderately dependent individuals showed a greater level of fear during a control sound test and then reacted similarly to independent horses in the social motivation sound test, we may conclude that the strong need to stay in proximity to members of the group mitigates fear caused by a frightening stimulus. As reported by Burattini et al. (2020), boldness and independence are two important behavioural traits that influence horses' fearfulness, assertiveness and sociability. Socially dependent horses are not at ease when left alone, away from the herd. Instead, they rely more on their conspecifics (Burattini et al. 2020). However, it should be noted that sound tests used in our study differed in the nature of the motivational conflict. In the non-social motivation test horses had to choose between staying and eating attractive food or fleeing (stay-avoidance conflict), whereas in social motivation test they had to decide whether to continue moving forward towards the herd or to stop/turn back to avoid the frightening stimulus (moving forward-avoidance conflict). Also, unlike in the test with food, the horses had no reason to stay near the loudspeaker once they had passed it. The mentioned differences between two tests may be the reason for limited correlations between horses’ responses to sound playback in different motivational conflicts. Lansade et al. (2008c) showed that within each sense, the greater the horses’ response to one stimulus, the greater their response to the other. However, as it was shown in our study, it is important to regard the context of presenting a testing stimulus during behavioural observations.

In the current study, also mares, that were shown to be more socially dependent than geldings, were definitely more anxious during a control sound test but reacted the same as the geldings during the sound test in the social motivation context. Thus, willingness to be reunited with conspecifics reduced the effect of a frightening stimulus. The only difference concerned behaviour after completion of a test, when the horses were waiting to join the herd. Again, the mares were more agitated, similar to the social isolation test (social dependence test). Lansade et al. (2022) noted that weaned horses still preferred their dam as well as other mares from their natal group after five months of separation, but this tendency was stronger in fillies than in colts. A stronger need to stay in a group in the case of mares may be due to the social structure of the species. In the wild, horses lived in groups comprising mainly females, and this is probably why social support is particularly important for them (Górecka-Bruzda et al. 2022). However, it should be noted that the geldings may have reacted differently than it would be seen under natural conditions in stallions.

The results of the current study may have practical implications. Horse owners should be aware of the existence of social dependence in horses and its correlations with fearfulness. However, similarly to Christensen et al. (2008) and Ricci‑Bonot et al. (2021), we confirmed the role of conspecifics as a social buffer and the importance of social motivation to confront a stressor. For example, in the study of Dai et al. (2019), horses were taught loading into a trailer in groups, which allowed their gregarious nature to be used in the learning process. We conclude that this factor should be considered in horse management when handling or schooling animals.

## Conclusions

Horses are highly motivated to stay close to their conspecifics, but they differ in their responses to social isolation. Socially dependent horses are not at ease being left alone outside the herd, in contrast to those more independent. However, while on the paddock, they engage in social interactions in a similar way. Dependent individuals are generally more fearful than independent animals. However, when facing sudden, novel audio stimulus in a social motivation context (motivation to join a herd), they react similarly to more self-reliant horses. Thus, social motivation may mitigate fear caused by a frightening event in horses. This finding should be taken into account in horse training and management.

## Data Availability

The datasets generated during and/or analysed during the current study are available from the corresponding author on reasonable request.
